# Sequences of Seven Complete Genomes of Human Parvovirus B19

**DOI:** 10.1128/MRA.00885-18

**Published:** 2018-09-20

**Authors:** Yuhuan Qiu, Zehui Zhao, Jianming Qiu

**Affiliations:** a Department of Microbiology, Molecular Genetics and Immunology, University of Kansas Medical Center, Kansas City, Kansas, USA; b College of Life Sciences, Sichuan University, Chengdu, China; Indiana University Bloomington

## Abstract

We are reporting the sequences of seven complete genomes of parvovirus B19, which were extracted from human plasma specimens collected in the United States. The seven B19 genome sequences, which are 5,596 nucleotides long and include the inverted terminal repeats (ITRs), share an identity of 96.73%. Their ITRs can be grouped into two types that have two nucleotide mutations between each other.

## Abstract (precis)

We are reporting the sequences of seven complete genomes of parvovirus B19, which were extracted from human plasma specimens collected in the United States. The seven B19 genome sequences, which are 5,596 nucleotides long and include the inverted terminal repeats (ITRs), share an identity of 96.73%.

## ANNOUNCEMENT

Human parvovirus B19 (B19V) is a member of the genus Erythroparvovirus in the family *Parvoviridae* (1). B19V infection causes erythema infectiosum (fifth disease or “slapped cheek syndrome”) in children, aplastic crisis in sickle cell disease patients, and hydrops fetalis in pregnant women (2). B19V contains a single-stranded DNA (ssDNA) genome of approximately 5,600 nucleotides (nt), which includes two identical inverted terminal repeats (ITRs) that are 383 nucleotides long. A number of B19V strains have been isolated from clinical specimens and sequenced; however, only four complete B19V genome sequences have been reported (3–5). The B19V ITRs are essential to virus replication; therefore, information about the ITRs of these clinical B19V isolates is important for understanding the relationship between the genome variations and the disease outcomes. Therefore, we aimed to sequence the complete genome of B19V in clinical specimens.

Sixteen plasma specimens, which contained the B19V genome with more than 10^10^ copies/ml, were received from Viracor Eurofins Laboratories (Lee’s Summit, MO) and used to extract the viral DNA using the QIAamp DNA blood minikit (Qiagen). To sequence the region between the two ITRs, we amplified the viral DNA as five PCR fragments that span the viral genome of 5,000 nucleotides with overlaps between the amplified DNA fragments. The PCR-amplified DNA was directly sequenced on an ABI 3730XL sequencer at MCLAB (South San Francisco, CA). Due to fact that the ITR contains a hairpin DNA structure, we were unable to amplify it with PCR. Since B19V contains either a positive or negative ssDNA genome, we heated the viral DNA and annealed it. The annealed viral DNA was blunted and digested with AflII and PciI restriction enzymes, which were used to clone the left and right ITRs, respectively, following a published method (4). Positive clones were digested with BssHII, which is in the middle of the ITR. The extracted DNA fragments separated by electrophoresis on 1.5% agarose gel were then sequenced on an ABI 3730XL sequencer at MCLAB.

We processed the sequencing reads by trimming with a PCR adapter using FASTA tools and quality filtering using PRINSEQ. The MIRA 4 assembler software was used to perform *de novo* assembly. Finally, we obtained seven complete genome sequences. A comparison of the seven B19V genome sequences revealed that they share an identity of 96.73%. The amino acid identities of the large nonstructural protein (NS1) and the large structural protein (VP1) are 97.62% and 97.44%, respectively. Notably, there are four mutations in the ITR regions. The KU2, KU3, KU4, KU5, and KU11 isolates have ITR regions identical to those of the J35 isolate (GenBank accession no. AY386330) (4). KU8 and KU12 have mutations of G to A at nt 115 and C to T at nt 251 in the left ITR, and mutations of G to A at nt 5346 and C to T at nt 5473 in the right ITR. The two ITRs are identical in each genome (3). Thus, these results suggest that these seven B19V isolates belong to genotype 1 (6).

### Data availability.

Seven complete genome sequences of the B19V isolates, KU2, KU3, KU4, KU5, KU8, KU11, and KU12, have been deposited in GenBank under the accession no. KM393163, KM393164, KM393165, KM393166, KM393167, KM393168, and KM393169, respectively.
